# Incisor Molar Hypomineralization and Quality of Life: A Population-Based Study with Brazilian Schoolchildren

**DOI:** 10.1155/2021/6655771

**Published:** 2021-03-03

**Authors:** Liege Helena Freitas Fernandes, Isla Camila C. Laureano, Lunna Farias, Natália M. Andrade, Franklin Delano Soares Forte, Catarina Ribeiro Barros Alencar, Alessandro Leite Cavalcanti

**Affiliations:** ^1^Department of Dentistry, State University of Paraíba, Campina Grande, Paraíba, Brazil; ^2^Department of Clinical and Social Dentistry, Federal University of Paraíba, João Pessoa, Paraíba, Brazil; ^3^Academic Unit of Biological Sciences, Faculty of Dentistry, Federal University of Campina Grande, Patos, Paraíba, Brazil

## Abstract

**Aim:**

To assess the impact of incisor molar hypomineralization (MIH) on oral health-related quality of life (OHRQoL) according to the perception of students and their parents/caregivers.

**Materials and Methods:**

This is a cross-sectional population-based study with 463 Brazilian students aged 11–14 years. OHRQoL was measured using the Child Perceptions Questionnaire (B-CPQ_11-14_ISF: 16) applied to students and the short version of the Parental-Caregiver Perceptions Questionnaire (B-P-CPQ) applied to parents/caregivers. The diagnosis of MIH followed the European Academy of Paediatric Dentistry criteria modified in 2019. Caries experience (ICDAS II), malocclusion (DAI), and socioeconomic and demographic factors were assessed as confounding factors for impact on OHRQoL. Cluster analysis was carried out to dichotomize the negative impact into greater and lesser impact. The chi-square test and Poisson regression were performed (*p* < 0.05) to verify associations between quality of life and MIH, adjusted for confounding variables.

**Results:**

The prevalence of MIH was 10.8%. Multivariate regression demonstrated that caries experience was the only oral disease that impacted OHRQoL according to students' self-perception in the functional limitation domain (PR = 1.82; 95% CI = 1.20–2.77) and in the total questionnaire score (PR = 1.59; 95% CI = 1.00–2.51). However, according to the perception of parents/caregivers, in addition to caries experience, which affected OHRQoL in the oral symptoms (PR = 3.57; 95% CI = 1.71–7.414) and emotional well-being domains (PR = 1.71; 95% CI = 1.08–2.69), as well as in the total B-P-CPQ score (PR = 1.67; 95% CI = 1.01–2.76), malocclusion also affected OHRQoL in the social well-being domain (PR = 1.50; 95% CI = 1.07–2.10) and in the total questionnaire score (PR = 1.54; 95% CI = 1.11–2.15).

**Conclusion:**

According to students and their parents/caregivers' perception, incisor molar hypomineralization did not influence OHRQoL of the studied sample.

## 1. Introduction

Quality of life is defined as the perception of well-being and personal and subjective manifestation of good feeling within the sociocultural context in which one lives [1]. During childhood, oral changes can affect the quality of children's systemic health [2]. Additionally, the oral condition also influences the quality of life, which can greatly impact psychological and social aspects [1]. The negative effects of poor dental appearance are irrefutable, also affecting social interaction [3].

The literature reports that enamel defects affect the quality of life of individuals with this condition since enamel defects can modify both aesthetics and the function of teeth [1]. Among these changes, incisor molar hypomineralization (MIH) has stood out and received increasing attention from dentists in several countries [4].

MIH is a qualitative enamel defect of systemic nature affecting one or more permanent first molars, with or without incisor involvement [5]. Clinically, it can vary from the lightest forms, characterized by well-marked opacities to posterior ruptures [5], since in MIH-affected teeth, the enamel is porous and susceptible to fracture [6].

Teeth with MIH generally present greater sensitivity [7], greater tendency to develop carious lesions [8], difficulty in obtaining the anesthetic effect [6, 9], and require multiple clinical interventions due to the difficulty in obtaining satisfactory adhesion of restorative materials to the dental substrate [9, 10], in addition to longer clinical care [10]. Therefore, MIH is associated with a series of oral morbidities, such as tooth hypersensitivity and poor dental aesthetics, affecting the quality of life [11].

The association between the presence of MIH and quality of life has been previously reported in some studies both in Brazil [12, 13] and in other countries, such as Colombia [1] and Mexico [14]. However, given the existence of particularities in the occurrence and comorbidities associated with MIH [5, 8, 15, 16], this relationship needs to be investigated in more detail, considering its cultural and socioeconomic diversities [17].

Thus, in view of the scarcity of literature data, this study aimed to assess the impact of the presence and severity of MIH on the OHRQoL of Brazilian schoolchildren aged 11–14 years.

## 2. Materials and Methods

### 2.1. Study Design and Location

This is a cross-sectional study conducted in Campina Grande, Paraíba, Brazil. The city has an estimated population of 407,472 inhabitants, Human Development Index (HDI) of 0.72, and Gini coefficient of 0.58 [18]. This study followed recommendations established by the Strengthening the Reporting of Observational Studies in Epidemiology (STROBE) [19].

### 2.2. Population and Sample Calculation

Participants were selected from a total population of 53,596 schoolchildren [18], regularly enrolled in elementary schools in the municipality of Campina Grande, Brazil. Sampling was of probabilistic type by clusters, and for sample calculation, the formula of infinite population was used: *n* = *z*^2^ × *P* (1−*P*)/FE^2^, where *n* represents the sample size; *z* represents the confidence level (95%), standard deviation (1.96); *P* represents the expected prevalence of the phenomenon to be investigated, 18.4% [12]; FE represents the predicted sample error factor (5%). A correction factor of 1.8 was used, as well as an increment of 10% to compensate for possible losses, with the final sample estimated at 463 students. Two public schools were drawn in each of the six selected urban Health Districts (HD), totaling 12 institutions.

### 2.3. Inclusion and Exclusion Criteria

All 11–14-year-old schoolchildren who had all first permanent molars fully erupted were included. Schoolchildren with fixed orthodontic appliance at the time of evaluation were excluded [12–14], as well as those with special needs (according to parents' reports) who did not cooperate with a clinical examination or were unable to answer the questionnaires.

### 2.4. Calibration

Theoretical and practical calibrations of the three examiners were performed by gold standard researchers with previous experiences in epidemiological investigations to diagnose MIH [20], dental caries [20, 21], and malocclusion. For MIH, Cohen's Kappa coefficient was 0.61–0.72 for interexaminer calibration and 0.67–0.83 for intraexaminer calibration.

For dental caries was used the International Caries Detection and Assessment System (ICDAS II) index, examiners took theoretical training online at https://www.iccms-web.com/, and regarding discussions about clinical diagnosis [22], the e practical stage was held in a public school. The interexaminer Kappa correlation values found ranged from 0.80 to 0.90 and intraexaminers values from 0.71 to 0.75.

For malocclusion, criteria proposed by Jenny and Cons [23] and the Brazilian Ministry of Health [24] were used. Cohen's Kappa coefficient was 0.76–0.89 for interexaminer calibration and 0.82–0.94 for intraexaminer calibration.

### 2.5. Data Collection

Data collection was performed in two stages between September and December 2019. The first stage was performed by parents/caregivers. They signed the consent form and answered the socioeconomic questionnaire and the Brazilian short form of the Parental-Caregivers' Perceptions Questionnaire (P-CPQ) [25].

The social and demographic questionnaires contained questions concerning parents/caregivers and students such as sex, age, family income (categorized based on the Brazilian monthly minimum wage amount equivalent to US$ 264.00), parents/caregivers' schooling in years of formal study (≤8 years of study/>8 years of study), and family structure (nuclear family structure when the child lives with parents who were married or in a stable union and nonnuclear structure when the child lives with only one single, divorced, or widowed parent), in addition to data on the use of dental services.

The reduced form of the B-P-CPQ questionnaire has 13 questions divided into three domains: oral symptoms, functional limitations, and well-being, referring to the last three months. The answer options range from zero to four points (between never and every day or almost every day). “I do not know” responses are allowed and scored 0. The total score ranged from 0 to 52. The higher the score, the higher the impact of oral condition on quality of life [25].

The second stage was performed by the schoolchildren whose parents/caregivers consented to study participation. They were directed to answer the self-reported 16-item Child Perception Questionnaire (CPQ_11–14_ISF:16) [26] and were clinically assessed.

The Brazilian version of the questionnaire CPQ_11-14_ISF:16 consisted of 16 items divided into four areas: oral symptom, functional limitation, emotional well-being, and social well-being. Each item addressed the frequency of events in the previous three months. Response options ranged from zero to four points, indicating the occurrence frequency (between “never” and “every day or almost every day”). The total score ranged from 0 to 64, and higher scores denoted a more negative impact of oral conditions on OHRQoL [26].

Before the clinical examination, children were instructed on oral hygiene procedures and received fluoridated toothpaste and toothbrush by means of which their teeth were cleaned by supervised brushing.

Clinical examinations took place in a reserved place in the school, under natural light, with the help of headlamps (JWS Lanternas, São Paulo, SP, Brasil). Researchers used all personal protective equipment, mouth mirrors (Golgran Indústria e Comércio de Instrumental Odontológico, São Caetano do Sul, SP, Brazil), and WHO probes (Trinity Indústria e Comércio Ltda., São Paulo, SP, Brazil) and sterilized in autoclave gauze (Gnatus Equipamentos Médico-Odontológicas Ltda., Barretos, SP, Brasil) to dry teeth, which is in line with infection control standards [27].

The presence of MIH was recorded when at least one first permanent molar was affected by demarcated opacities, ranging from white-cream-yellow-brown, due to posteruptive enamel fractures, by atypical restorations/atypical carious lesions, or when there was an absence of permanent molars due to MIH—all these clinical features with or without the involvement of incisors [20]. MIH severity was classified as mild, only color changes—cream, white, yellow, orange, or brown—and severe—fracture and/or atypical restoration/atypical caries/loss due to MIH [21]. MIH severity was defined by the most severe defect observed in first permanent molars and/or permanent incisors [28].

Schoolchildren were also evaluated to determine their caries experience, malocclusion, and presence of other enamel defects, as they are considered confounding variables [12]. Children were evaluated to determine their dental caries experience according to the International Caries Detection and Assessment System II (ICDAS II) [29]. Dental caries was considered present for ICDAS code >0. Malocclusion was assessed using the Dental Aesthetic Index (DAI). DAI components are divided into three groups: tooth, space, and occlusion and placed in an equation, which classified as absence (DAI ≤25) and presence (DAI >25) of malocclusion [23].

Differential MIH diagnosis was performed with diffuse opacities (fluorosis), with white spots of dental caries, imperfect amelogenesis, enamel hypoplasia, and hypomineralization defects other than MIH [20].

### 2.6. Statistical Analysis

Data were analyzed using IBM SPSS Statistics (IBM SPSS Statistics for Windows, Version 22.0. Armonk, NY: IBM Corp). The internal consistency of the OHRQoL questionnaire was examined by computing Cronbach's alpha coefficients. In order to dichotomize the total score and the domains of the B-CPQ_11-14_ISF:16 and B-P-CPQ instruments in a greater and lesser negative impact on OHRQoL, *k*-means cluster analysis was performed. Cluster analysis assesses the pattern of responses for each item separately and for the formation of clusters. It considers the correlation between the responses to the instrument and can be valid because there is no cutoff pattern for the sum of questions of B-CPQ_11-14_ISF:16 and B-P-CPQ instruments for the total score and their domains.

A descriptive statistical analysis was performed, and the Kolmogorov–Smirnov test was used to verify the normality of quantitative variables. The chi-square test was used to verify the association between the negative impact on OHRQoL with MIH and confounding variables (caries experience, malocclusion, other enamel defects, and socioeconomic factors). In multivariate analysis, Poisson regression was performed with a robust variance; all variables with *p* < 0.2 in the bivariate analysis were included in the adjusted multivariate analysis. Prevalence ratios (PR) with the respective 95% confidence intervals (95% CI) were calculated for associations. Variable “MIH in molars” was removed from the adjusted analysis of the B-P-CPQ questionnaire for presenting collinearity with variable “Presence of MIH.” A significance level of 5% was adopted.

### 2.7. Ethical Aspects

This study was approved by the local institutional ethics committee under opinion No. 3.155.847. All study procedures were conducted in accordance with Resolution 466/12 of the Brazilian National Health Council and the Declaration of Helsinki and its subsequent amendments.

## 3. Results

A total of 590 children were invited, of whom 463 agreed to participate in the study. The prevalence of MIH was 10.8% (*n* = 50). Regarding severity, 22 (44.0%) students had a mild degree and 28 (56.0%) had a severe degree. A total of 80.1% of the sample had caries experience, 48.4% had malocclusion, and with respect to other enamel defects, 10.6% was diagnosed with fluorosis, 1.1% with hypoplasia, 0.2% had imperfect amelogenesis, and 4.5% had other types of hypomineralization (Table 1).

Tables 2 and 3 show the bivariate analysis between the negative impact of domains and the total score of OHRQoL instruments and independent variables. It was observed that according to the self-perception of students, the presence of MIH, regardless of severity degree, was not associated with OHRQoL. Regarding parents/caregivers' perception, schoolchildren with MIH were associated with a negative impact on OHRQoL in the emotional well-being domain (*p*=0.046). In addition, the presence of MIH in molars was also associated with the emotional well-being domain (*p*=0.046).

Tables 4 and 5 show the crude multivariate models, and Tables 6 and 7 show the final multivariate models of negative impacts of independent variables on self-perceived OHRQoL and OHRQoL perceived by parents, distributed by domains and total scores of the B-CPQ_11-14_ISF: 16 and B-P-CPQ instruments. According to the self-perception of students, the only oral disease that had influence on OHRQoL was dental caries experience, which had 82.8% greater impact rate in the functional limitation domain (PR_adjusted_ = 1.82; 95% CI = 1.20–2.77) and 59% in the total B-CPQ_11-14_ISF:16 score (PR_adjusted_ = 1.59; 95% CI = 1.00–2.51). In addition, females perceived greater impact on OHRQoL in the functional limitation domain (PR_adjusted_ = 1.52; 95% CI = 1.14–2.02), and the lower parental schooling had impact on emotional well-being (PR_adjusted_ = 1.59; 95% CI = 1.17–2.15) and social well-being domains (PR_adjusted_ = 1.45; 95% CI = 1.01–2.07) (Table 6).

According to the perception of parents/caregivers, students with caries experience had 57.5% greater impact rate in the oral symptoms domain (PR_adjusted_ = 3.57; 95% CI = 1.71–7.414), 71.1% in emotional well-being domain (PR_adjusted_ = 1.71; 95% CI = 1.08–2.69), and 67.8% in the total B-P-CPQ score (PR_adjusted_ = 1.67; 95% CI = 1.01–2.76). Additionally, malocclusion also negatively influenced OHRQoL in the social well-being domain (PR_adjusted_ = 1.50; 95% CI = 1.07–2.10) and in the total questionnaire score (PR_adjusted_ = 1.54; 95% CI = 1.11–2.15). Female gender, older age, lower family income, and never having visited the dentist also had negative impact on quality of life (Table 7).

## 4. Discussion

MIH is an enamel defect that has been increasingly studied by the dental community [4]. However, literature is still scarce concerning the impact of MIH on patients' oral health quality. Thus, studies of this nature are necessary since they contribute to the planning of oral health promotion programs that are socially appropriate for the target population [17].

Although there is convincing evidence that children with enamel defects experience a variety of psychosocial impacts [3], which can affect their quality of life and cause behavioral problems [6], in this study, the presence of MIH or other enamel defects did not demonstrate impact on OHRQoL from the self-perception of students or the perception of their parents/caregivers. In other studies conducted with Brazilian children and adolescents, MIH had a negative impact on OHRQoL [12, 13]. It is believed that the divergence among studies may have occurred due to the cultural differences of the Brazilian population since Brazil is a country with continental dimensions with different cultures depending on the region [30, 31]. When considering culturally diverse individuals, it is known that in addition to their different physical, emotional, or social needs, their oral health perceptions may also differ [32].

In this study, caries experience had a higher prevalence when compared to MIH. The occurrence of this disease has been decreasing in recent decades, causing many Western populations to be classified as having a low caries rate; however, its control remains a challenge for many population groups [33]. The literature has shown that caries' presence negatively affects the OHRQoL of patients [2, 34, 35], which was also observed in this study. This negative impact possibly masked the influence of MIH on the OHRQoL of the population under study.

Dental caries cause functional changes, such as chewing and speaking problems, difficulty sleeping and irritability, also influencing other factors, such as school absenteeism [2]. These aspects may explain the impact of this condition on the OHRQoL of students in this study, both in their self-perception and in the perception of their parents/caregivers, thus evidencing the harmful effect of caries on the oral health of students [34].

Malocclusion had a negative impact on OHRQoL from the perception of parents/caregivers. This finding is in line with results found in the literature [12, 36], which was expected considering that, in this age group, corresponding to the beginning of adolescence, social life becomes more intense and appearance tends to become more important [36].

In the present study, female students showed a higher prevalence of impact on OHRQoL in the functional limitation domain of CPQ_11-14_ ISF:16 and the social well-being and total B-P-CPQ score from the perception of parents/caregivers. This finding is similar to that reported in other studies [12–14]. Women are more concerned than men about oral health perception [37].

Oral diseases are cumulative and tend to worsen with increasing age [2], justifying the relationship between older ages and greater impact on the social well-being domain of OHRQoL from the perception of parents/caregivers. Thus, the diagnosis and treatment of oral changes should occur as early as possible, reducing impacts on students' quality of life [2].

Concerning family income, the reduced availability of access to oral health services is a common reality in populations with low levels of financial resources [14]. Since Brazil is a country with great social disparities, this fact may explain the association between lower family income and a negative impact on the functional limitation of B-P-CPQ on the quality of life of students. This relationship has also been observed in other studies [12, 13, 34].

The lower the schooling, the greater the chances of presenting inadequate oral health [37, 38]. This statement may clarify the association between low schooling and impact on the emotional and social well-being domains of the OHRQoL self-perception questionnaire found in this study. Schooling may also reflect knowledge about the importance and maintenance of healthy oral habits, as well as being strongly associated with oral self-care [37, 39].

The cross-sectional design is among the limitations of this study, which analyzes data related to the perceptions of a specific moment [12], not allowing causal inferences. However, the population representativeness through sample calculation, high inter-examiner reliability, the conduction of a pilot study, and the use of validated questionnaires with a high response rate can be highlighted as strengths, aspects that reinforce the study validity.

For the development of public oral health policies from the perspective of a broad and appropriate health concept focused on improving quality of life, it is essential to understand the particularities of a given population [32]. Thus, the relevance of studies on the perception of OHRQoL in this planning is emphasized, as these strategies must be planned, taking into account the opinion of students and their parents/caregivers.

## 5. Conclusion

MIH had no impact on the OHRQoL of schoolchildren aged 11–14 years from the self-perception of schoolchildren or their parents/caregivers.

## Figures and Tables

**Table 1:** Sample characterization regarding sociodemographic, economic, and clinical factors.

Variable	*n* (%)
Sex	
Female	293 (63.3)
Male	170 (36.7)

Age	
11	141 (30.5)
12	168 (36.3)
13	101 (21.8)
14	53 (11.4)

Family income in minimum wages (MW)^1^	
≤1 MW	350 (83.7)
>1 MW	68 (16.3)

Parents/caregivers' schooling	
≤8 years of study	276 (60.7)
>8 years of study	179 (39.3)

Family structure	
Nonnuclear	229 (49.5)
Nuclear	234 (50.5)

Visited the dentist once in life	
Yes	356 (76.9)
No	107 (23.1)

MIH	
Present	50 (10.8)
Absent	413 (89.2)

MIH on incisors	
Present	16 (3.5)
Absent	447 (96.5)

MIH on molars	
Present	50 (10.8)
Absent	413 (89.2)

MIH severity	
Mild	22 (44.0)
Severe	28 (56.0)

Dental caries experience	
Yes	371 (80.1)
No	92 (19.9)

Malocclusion	
Present	224 (48.4)
Absent	239 (51.6)

Dental fluorosis	
Present	49 (10.6)
Absent	414 (89.4)

Hypoplasia^2^	
Present	5 (1.1)
Absent	458 (98.9)

Amelogenesis imperfecta^2^	
Present	1 (0.2)
Absent	462 (99.8)

Hypomineralization other than MIH	
Present	21 (4.5)
Absent	442 (95.5)

^1^Value of the Brazilian minimum wage in force at the time of the research was equivalent to Rs. 99,800 (US$ 264.00). ^2^Due to the very low *N*, the variable was not included in the regression analysis.

**Table 2:** Association between domains and the total score of the B-CPQ_11-14_ ISF:16 instrument with MIH and confounding factors.

	Domains														
	Oral symptoms			Functional limitation			Emotional well-being			Social well-being			Total score		
	Greater *n* (%)	Lesser *n* (%)	*p* value^*∗*^	Greater *n* (%)	Lesser *n* (%)	*p* value^*∗*^	Greater *n* (%)	Lesser *n* (%)	*p* value^*∗*^	Greater *n* (%)	Lesser *n* (%)	*p* value^*∗*^	Greater *n* (%)	Lesser *n* (%)	*p* value^*∗*^
MIH															
Present	26 (52.0)	24 (48.0)	0.814	21 (42.0)	29 (58.0)	0.241	14 (28.0)	36 (72.0)	0.569	7 (14.0)	43 (86.0)	0.075+	12 (24.0)	38 (76.0)	0.589
Absent	222 (53.8)	191 (46.2)		139 (33.7)	274 (66.3)		132 (32.0)	281 (68.0)		105 (25.4)	308 (74.6)		114 (27.6)	299 (72.4)	

MIH severity															
Mild	10 (45.5)	12 (54.5)	0.412	10 (45.5)	12 (54.5)	0.661	4 (18.2)	18 (81.8)	0.171+	3 (13.6)	19 (86.4)	0.948	3 (13.6)	19 (86.4)	0.128+
Severe	16 (57.1)	12 (42.9)		11 (39.3)	17 (60.7)		10 (35.7)	18 (64.3)		4 (14.3)	24 (85.7)		9 (32.1)	19 (67.9)	

MIH on incisors															
Yes	7 (43.8)	9 (56.2)	0.423	4 (25.0)	12 (75.0)	0.413	3 (18.8)	13 (81.2)	0.263	1 (6.2)	15 (93.8)	0.088+	1 (6.2)	15 (93.8)	0.055+
No	241 (53.9)	206 (46.1)		156 (34.9)	291 (65.1)		143 (32.0)	304 (68.0)		111 (24.8)	336 (75.2)		125 (28.0)	322 (72.0)	

MIH on molars															
Yes	26 (52.0)	24 (48.0)	0.814	21 (42.0)	29 (58.0)	0.241	14 (28.0)	36 (72.0)	0.569	7 (14.0)	43 (86.0)	0.075+	12 (24.0)	38 (76.0)	0.589
No	222 (53.8)	191 (46.2)		139 (33.7)	274 (66.3)		132 (32.0)	281 (68.0)		105 (25.4)	308 (74.6)		114 (27.6)	299 (72.4)	

Dental caries experience															
Yes	207 (55.8)	164 (44.2)	0.053+	141 (38.0)	230 (62.0)	**0.002+**	125 (33.7)	246 (66.3)	**0.045+**	95 (25.6)	276 (74.4)	0.153+	109 (29.4)	262 (70.6)	**0.035+**
No	41 (44.6)	51 (55.4)		19 (20.7)	73 (79.3)		21 (22.8)	71 (77.2)		17 (18.5)	75 (81.5)		17 (18.5)	75 (81.5)	

Malocclusion															
Present	121 (54.0)	103 (46.0)	0.850	76 (33.9)	148 (66.1)	0.783	79 (35.3)	145 (64.7)	0.094+	62 (27.7)	162 (72.3)	0.090+	65 (29.0)	159 (71.0)	0.398
Absent	127 (53.1)	112 (46.8)		84 (35.1)	155 (64.9)		67 (28.0)	172 (72.0)		50 (20.9)	189 (79.1)		61 (25.5)	178 (74.5)	

Dental fluorosis															
Present	22 (44.9)	27 (55.1)	0.198+	17 (34.7)	32 (65.3)	0.983	15 (30.6)	34 (69.4)	0.883	13 (26.5)	36 (73.5)	0.686	15 (30.6)	34 (69.4)	0.572
Absent	226 (54.6)	188 (45.5)		143 (34.5)	271 (65.6)		131 (31.6)	283 (68.4)		99 (23.9)	315 (76.1)		111 (26.8)	303 (73.2)	

Hypomineralization other than MIH															
Present	12 (57.1)	9 (42.9)	0.736	6 (28.6)	15 (71.4)	0.555	6 (28.6)	15 (71.4)	0.765	5 (23.8)	16 (76.2)	0.967	6 (28.6)	15 (71.4)	0.886
Absent	236 (53.4)	206 (46.6)		154 (34.8)	288 (65.2)		140 (31.7)	302 (31.7)		107 (24.2)	335 (75.8)		120 (27.1)	322 (72.9)	

Sex															
Female	153 (52.2)	140 (47.8)	0.446	116 (39.6)	177 (60.4)	**0.003+**	97 (33.1)	196 (66.9)	0.339	70 (23.9)	223 (76.1)	0.843	84 (28.7)	209 (71.3)	0.356
Male	95 (55.9)	75 (44.1)		44 (25.9)	126 (74.1)		49 (28.8)	121 (71.2)		42 (24.7)	128 (75.3)		42 (24.7)	128 (75.3)	

Age															
11	77 (54.6)	64 (45.4)	0.928	54 (38.3)	87 (61.7)	0.694	45 (31.9)	96 (68.1)	0.895	33 (23.4)	108 (76.6)	0.441	41 (29.1)	100 (70.9)	0.668
12	89 (53.0)	79 (47.0)		54 (32.1)	114 (67.9)		51 (30.4)	117 (69.6)		35 (20.8)	133 (79.2)		41 (24.4)	127 (75.6)	
13	52 (51.5)	49 (48.5)		35 (34.7)	66 (65.3)		31 (30.7)	70 (69.3)		29 (28.7)	72 (71.3)		27 (26.7)	74 (73.3)	
14	30 (56.6)	23 (43.4)		17 (32.1)	36 (67.9)		19 (35.8)	34 (64.2)		15 (28.3)	38 (71.7)		17 (32.1)	36 (67.9)	

Family income in minimum wages (MW)															
≤1 MW	187 (53.4)	163 (46.6)	0.882	122 (34.9)	228 (65.1)	0.527	107 (30.6)	243 (69.4)	0.442	87 (24.9)	263 (75.1)	0.816	94 (26.9)	256 (73.1)	0.496
>1 SW	37 (54.4)	31 (45.6)		21 (30.9)	47 (69.1)		24 (35.3)	44 (64.7)		16 (23.5)	52 (76.5)		21 (30.9)	47 (69.1)	

Parents/caregivers' schooling															
≤8 years of study	148 (53.6)	128 (46.4)	0.906	100 (36.2)	176 (63.8)	0.402	103 (37.3)	173 (62.7)	**0.002+**	76 (27.5)	200 (72.5)	**0.038+**	85 (30.8)	191 (69.2)	**0.049+**
>8 years of study	97 (54.2)	82 (45.8)		58 (32.4)	121 (67.6)		42 (23.5)	137 (76.5)		34 (19.0)	145 (81.0)		40 (22.3)	139 (77.7)	

Family structure															
Nonnuclear	130 (56.8)	99 (43.2)	0.171+	78 (34.1)	151 (65.9)	0.824	73 (31.9)	156 (68.1)	0.875	52 (22.7)	177 (77.3)	0.461	62 (27.1)	167 (72.9)	0.947
Nuclear	118 (50.4)	116 (49.6)		82 (35.0)	152 (65.0)		73 (31.2)	161 (68,8)		60 (25.6)	174 (74.4)		64 (27.4)	170 (72.6)	

Visit to the dentist															
Yes	193 (54.2)	163 (45.8)	0.609	130 (36.5)	226 (63.5)	0.106+	112 (31.5)	244 (68.5)	0.951	86 (24.2)	270 (75.8)	0.976	98 (27.5)	258 (72.5)	0.782
No	55 (51.4)	52 (48.6)		30 (28.0)	77 (72.0)		34 (31.8)	73 (68.2)		26 (24.3)	81 (75.7)		28 (26.2)	79 (73.8)	

^*∗*^Chi-square test + variables with *p* < 0.20 included in the adjusted multivariate model. ^+^Statistically significant.

**Table 3:** Association between domains and the total score of the B-P-CPQ instrument with MIH and confounding factors.

	Domains														
	Oral symptoms			Functional limitation			Emotional well-being			Social well-being			Total score		
	Greater *n* (%)	Lesser *n* (%)	*p* value^*∗*^	Greater *n* (%)	Lesser *n* (%)	*p* value^*∗*^	Greater *n* (%)	Lesser *n* (%)	*p* value^*∗*^	Greater *n* (%)	Lesser *n* (%)	*p* value^*∗*^	Greater *n* (%)	Lesser *n* (%)	*p* value^*∗*^
MIH															
Present	17 (34.0)	33 (66.0)	0.072+	15 (30.0)	35 (70.0)	0.805	21 (42.0)	29 (58.0)	**0.046+**	12 (24.00)	38 (76.0)	0.844	15 (30.0)	35 (70.0)	0.272
Absent	93 (22.5)	320 (77.5)		131 (31.7)	282 (68.3)		117 (28.3)	296 (71.7)		94 (22.8)	319 (77.2)		95 (23.0)	318 (77.0)	
MIH severity															
Mild	7 (31.8)	15 (68.2)	0.773	4 (18.2)	18 (81.8)	0.106+	7 (31.8)	15 (68.2)	0.196+	4 (18.2)	18 (81.8)	0.393	4 (18.2)	18 (81.8)	0.106+
Severe	10 (35.7)	18 (64.3)		11 (39.3)	17 (60.7)		14 (50.0)	14 (50.0)		8 (28.6)	20 (71.4)		11 (39.3)	17 (60.7)	
MIH on incisors															
Yes	4 (25.0)	12 (75.0)	0.905	3 (18.8)	13 (81.2)	0.263	7 (43.8)	9 (56.2)	0.215	1 (6.2)	15 (93.8)	0.107+	4 (25.0)	12 (75.0)	0.905
No	106 (23.7)	341 (76.3)		143 (32.0)	304 (68.0)		131 (29.3)	316 (70.0)		105 (23.5)	342 (76.5)		106 (23.7)	341 (76.3)	
MIH on molars															
Yes	17 (34.0)	33 (66.0)	0.072+	15 (30.0)	35 (70.0)	0.805	21 (42.01)	29 (58.0)	**0.046+**	12 (24.0)	38 (76.0)	0.844	15 (30.0)	35 (70.0)	0.272
No	93 (22.5)	320 (77.5)		131 (31.7)	282 (68.3)		117 (28.3)	296 (71.7)		94 (22.8)	319 (77.2)		95 (23.0)	318 (77.0)	
Dental caries experience															
Yes	103 (27.8)	268 (72.2)	**0.000+**	124 (33.4)	247 (66.6)	0.079+	121 (32.6)	250 (67.4)	**0.008+**	90 (24.3)	281 (75.7)	0.160+	96 (25.9)	275 (74.1)	0.032+
No	7 (7.6)	85 (92.4)		22 (23.9)	70 (76.1)		17 (18,5)	75 (81,5)		16 (17,4)	76 (82,6)		14 (15.2)	78 (84.8)	
Malocclusion															
Present	59 (26.3)	165 (73.7)	0.206	78 (34.8)	146 (65.2)	0.140+	71 (31.7)	153 (68.3)	0.389	63 (28.1)	161 (71.9)	**0.010+**	66 (29.5)	158 (70.5)	**0.005+**
Absent	51 (21.3)	188 (78.7)		68 (28.5)	171 (71.5)		67 (28.0)	172 (72.0)		43 (18.0)	196 (82.0)		44 (18.4)	195 (81.6)	
Dental fluorosis															
Present	11 (22.4)	38 (77.6)	0.820	13 (26.5)	36 (73.5)	0.425	15 (30.6)	34 (69.4)	0.896	14 (28.6)	35 (71.4)	0.317	13 (26.5)	36 (73.5)	0.630
Absent	99 (23.9)	315 (76.1)		133 (32.1)	281 (67.9)		123 (29.7)	291 (70.3)		92 (22.2)	322 (77.8)		97 (23.4)	317 (76.6)	
Hypomineralization other than MIH															
Present	3 (14.2)	18 (85.7)	0.297	6 (28.6)	15 (71.4)	0.765	4 (19.0)	17 (81.0)	0.270	4 (19.0)	17 (81.0)	0.668	5 (23.8)	16 (76.2)	0.995
Present	107 (24.2)	335 (75.8)		140 (31.7)	302 (68.3)		134 (30.3)	308 (69.7)		102 (23.1)	340 (76.9)		105 (23.8)	337 (76.2)	
Sex															
Female	84 (28.7)	209 (71.3)	**0.001+**	98 (33.4)	195 (66.6)	0.245	94 (32.1)	199 (67.9)	0.160+	78 (26.6)	215 (73.4)	**0.012+**	83 (28.3)	210 (71.7)	**0.002+**
Male	26 (15.3)	144 (84.7)		48 (28.2)	122 (71.8)		44 (25.9)	126 (74.1)		28 (16.5)	142 (83.5)		27 (15.9)	143 (84.1)	
Age															
11	32 (22.7)	109 (77.3)	0.839	49 (34.8)	92 (65.2)	0.400	45 (31.9)	96 (68.1)	0.392	30 (21.3)	111 (78.7)	**0.001+**	38 (27.0)	103 (73.0)	0.163+
12	40 (23.8)	128 (76.2)		45 (26.8)	123 (73.2)		44 (26.2)	124 (73.8)		27 (16.1)	141 (83.9)		30 (17.9)	138 (82.1)	
13	27 (26.7)	74 (73.3)		33 (32.7)	68 (67.3)		29 (28.7)	72 (71.3)		27 (27.7)	74 (73.3)		27 (26.7)	74 (73.3)	
14	11 (20.8)	42 (79.2)		19 (35.8)	34 (64.2)		20 (37.7)	33 (62.3)		22 (41.5)	31 (58.5)		15 (28.3)	38 (71.7)	
Family income in minimum wages (MW)															
≤1 MW	85 (24.3)	265 (75.7)	0.702	120 (34.3)	230 (65.7)	**0.027+**	110 (31.4)	240 (68.6)	0.194+	87 (24.9)	263 (75.1)	0.122+	90 (25.7)	260 (74.3)	0.248
>1 SW	18 (26.5)	50 (73.5)		14 (20.6)	54 (79.4)		16 (23.4)	52 (76.5)		11 (16.2)	57 (83.8)		13 (19.1)	55 (80.9)	
Parents/caregivers' schooling															
≤8 years of study	64 (23.2)	212 (76.8)	0.541	94 (34.1)	182 (65.9)	0.134+	82 (29.7)	194 (70.3)	0.917	72 (26.1)	204 (73.9)	0.058+	73 (26.4)	203 (73.6)	0.091+
>8 years of study	46 (25.7)	133 (74.3)		49 (27.4)	130 (72.6)		54 (30.2)	125 (69.8)		33 (18.4)	146 (81.6)		35 (19.6)	144 (80.4)	
Family structure															
Nonnuclear	55 (24.0)	174 (76.0)	0.897	77 (33.6)	152 (66.4)	0.338	70 (30.6)	159 (69.4)	0.723	56 (24.5)	173 (75.5)	0.429	56 (24.5)	173 (75.5)	0.728
Nuclear	55 (23.5)	179 (76.5)		69 (29.5)	165 (70.5)		68 (29.1)	166 (70.9)		50 (21.4)	184 (78.6)		54 (23.1)	180 (76.9)	
Visit to the dentist															
Yes	93 (26.1)	263 (73.9)	**0.029+**	111 (31.2)	245 (68.8)	0.765	112 (31.5)	244 (68.5)	0.156+	84 (23.6)	272 (76.4)	0.512	89 (25.0)	267 (75.0)	0.252
No	17 (15.9)	90 (84.1)		35 (32.7)	72 (67.3)		26 (24.3)	81 (75.7)		22 (20.6)	85 (79.4)		21 (19.6)	86 (80.4)	

^*∗*^Chi-square test + variables with *p* < 0.20 included in the adjusted multivariate model. ^+^Statistically significant.

**Table 4:** The crude multivariate model of the association between domains and the total score of the B-CPQ_11-14_ ISF:16 instrument with MIH and confounding factors.

	Domains									
	Oral symptoms		Functional limitation		Emotional well-being		Social well-being		Total score	
	PRg (95% CI)	*p* value	PRg (95% CI)	*p* value	PRg (95% CI)	*p* value	PRg (95% CI)	*p* value	PRg (95% CI)	*p* value
MIH										
Present							0.551 (0.272–1.116)	0.098		
Absent							1			

MIH severity										
Severe					1.964 (0.711–5.427)	0.193			2.357 (0.723–7.681)	0.155
Mild					1				1	

MIH on incisors										
Yes							0.252 (0.037–1.690)	0.156	0.224 (0.033–1.500)	0.123
No							1		1	

MIH on molars										
Yes							0.551 (0.272–1.116)	0.098		
No							1			

Dental caries experience										
Yes	1.252 (0.980–1.600)	0.072	**1.840 (1.208–2.804)**	**0.005**	1.476 (0.988–2.206)	0.058	1.386 (0.872–2.202)	0.167	**1.590 (1.006–2.512)**	**0.047**
No	1		1		1		1		1	

Malocclusion										
Present					1.258 (0.961–1.648)	0.095	1.323 (0.956–1.831)	0.091		
Absent					1		1			

Dental fluorosis										
Present	0.822 (0.591–1.135)	0.235								
Absent	1									

Sex										
Female			**1.530 (1.143–2.046)**	**0.004**						
Male			1							

Parents/caregivers' schooling										
≤8 years of study					**1.590 (1.172–2.159)**	**0.003**	**1.450 (1.013–2.074)**	**0.042**	1.378 (0.995–1.908)	0.053
>8 years of study					1		1		1	

Family structure										
Nonnuclear	1.126 (0.950–1.334)	0.172								
Nuclear	1									

Visit to the dentist										
No			0.768 (0.550–1.071)	0.120						
Yes			1							

Variables in bold correspond to those with *p* values <0.05 in the final model. Crude PR, crude prevalence ratio; 95% CI, 95% confidence interval.

**Table 5:** The crude multivariate model of the association between domains and the total score of the B-P-CPQ instrument with MIH and confounding factors.

	Domains									
	Oral symptoms		Functional limitation		Emotional well-being		Social well-being		Total score	
	PRg (95% CI)	*p* value	PRg (95% CI)	*p* value	PRg (95% CI)	*p* value	PRg (95% CI)	*p* value	PRg (95% CI)	*p* value
MIH										
Present	1.510 (0.987–2.311)	0.058			**1.483 (1.034–2.125)**	**0.032**				
Absent	1				1					

MIH severity										
Severe			2.161 (0.796–5.867)	0.131	1.571 (0.769–3.213)	0.215			2.161 (0.796–5.867)	0.131
Mild			1		1				1	

MIH on incisors										
Yes							0.266 (0.040–1.788)	0.173		
No							1			

MIH on molars										
Yes	1.510 (0.987–2.311)	0.058			**1.483 (1.034–2.125)**	**0.032**				
No	1				1					

Dental caries experience										
Yes	**3.649 (1.757–7.577)**	**0.001**	1.398 (0.945–2.068)	0.094	**1.765 (1.122–2.778)**	**0.014**	1.395 (0.863–2.255)	0.174	**1.700 (1.019–2.838)**	**0.042**
No	1		1		1		1		1	

Malocclusion										
Present			1.224 (0.935–1.602)	0.141			**1.563 (1.110–2.201)**	**0.010**	**1.600 (1.145–2.238)**	**0.006**
Absent			1				1		1	

Sex										
Female					1.240 (0.915–1.680)	0.166	**1.616 (1.096–2.383)**	**0.015**	**1.784 (1.206–2.637)**	**0.004**
Male					1		1		1	

Age										
14							**1.951 (1.243–3.061)**	**0.004**	1.050 (0.632–1.744)	0.850
13							1.256 (0.799–1.976)	0.323	0.992 (0.650–1.513)	0.970
12							0.755 (0.472–1.208)	0.241	0.663 (0.434–1.012)	0.057
11							1		1	

Family income in minimum wages (MW)										
≤1 MW			**1.665 (1.021–2.715)**	**0.041**	1.336 (0.847–2.107)	0.213	1.537 (0.868–2.720)	0.140		
>1 SW			1		1		1			

Parents/caregivers' schooling										
≤8 years of study			1.244 (0.931–1.662)	0.139			1.415 (0.981–2.042)	0.063	1.353 (0.947–1.932)	0.097
>8 years of study			1				1		1	

Visit to the dentist										
No	**0.608 (0.380–0.973)**	**0.038**			0.772 (0.535–1.116)	0.169				
Yes	1				1					

Variables in bold correspond to those with *p* values <0.05 in the final model. Crude PR, crude prevalence ratio; 95% CI, 95% confidence interval.

**Table 6:** The final multivariate model of the association between domains and the total score of the B-CPQ_11-14_ ISF: 16 instrument with MIH and confounding factors.

	Domains									
	Oral symptoms		Functional limitation		Emotional well-being		Social well-being		Total score	
	PRa (95% CI)	*p* value	PRa (95% CI)	*p* value	PRa (95% CI)	*p* value	PRa (95% CI)	*p* value	PRa (95% CI)	*p* value
Dental caries experience										
Yes			**1.828 (1.206–2.772)**	0.004					**1.590 (1.006–2.512)**	**0.047**
No			1						1	

Sex										
Female			**1.521 (1.141–2.028)**	0.004						
Male			1							

Parents/caregivers' schooling										
≤ 8 years of study					**1.590 (1.172–2.159)**	**0.003**	**1.450 (1.013–2.074)**	**0.042**		
>8 years of study					1		1			

B-CPQ_11-14_ ISF:16, Child Perceptions Questionnaire Brazilian version. Variables in bold correspond to those with *p* values <0.05 in the final model. Adjusted PR, adjusted prevalence ratio; 95% CI, 95% confidence interval.

**Table 7:** The final multivariate model of the association between domains and the total score of the B-P-CPQ instrument with MIH and confounding factors.

	Domains									
	Oral symptoms		Functional limitation		Emotional well-being		Social well-being		Total score	
	PRa (95% CI)	*p* value	PRa (95% CI)	*p* value	PRa (95% CI)	*p* value	PRa (95% CI)	*p* value	PRa (95% CI)	*p* value
MIH										
Present					1.398 (0.975–2.003)	0.068				
Absent					1					

MIH on molars¹										
Yes					-	-				
No					-					

Dental caries experience										
Yes	**3.575 (1.724–7.414)**	**0.001**			**1.711 (1.085–2.698)**	**0.021**			**1.678 (1.019–2.764)**	**0.042**
No	1				1				1	

Malocclusion										
Present							**1.502 (1.072–2.106)**	**0.018**	**1.547 (1.110–2.155)**	**0.010**
Absent							1		1	

Sex										
Female							**1.587 (1.086–2.320)**	**0.017**	**1.721 (1.167–2.539)**	**0.006**
Male							1		1	

Age										
14							**2.003 (1.293–3.104)**	**0.002**		
13							1.326 (0.847–2.075)	0.217		
12							0.789 (0.496–1.254)	0.315		
11							1			

Family income in minimum wages (MW)										
≤1 MW			**1.665 (1.021–2.715)**	**0.041**						
>1 SW			1							

Visit to the dentist										
No	0.632 (0.398–1.004)	0.052								
Yes	1									

B-P-CPQ, Parental-Caregiver Perceptions Questionnaire short Brazilian version. Variables in bold correspond to those with *p* values <0.05 in the final model. Adjusted PR, adjusted prevalence ratio; 95% CI, 95% confidence interval.

## Data Availability

The data used to support the findings of this study are included within the article.
